# Measuring specialization in species interaction networks

**DOI:** 10.1186/1472-6785-6-9

**Published:** 2006-08-14

**Authors:** Nico Blüthgen, Florian Menzel, Nils Blüthgen

**Affiliations:** 1 Department of Animal Ecology and Tropical Biology, University of Würzburg, Biozentrum, Am Hubland, 97074 Würzburg, Germany; 2 Institute of Theoretical Biology, Humboldt University, 10115 Berlin and Institute of Molecular Neurobiology, Free University of Berlin, 14195 Berlin, Germany

## Abstract

**Background:**

Network analyses of plant-animal interactions hold valuable biological information. They are often used to quantify the degree of specialization between partners, but usually based on qualitative indices such as 'connectance' or number of links. These measures ignore interaction frequencies or sampling intensity, and strongly depend on network size.

**Results:**

Here we introduce two quantitative indices using interaction frequencies to describe the degree of specialization, based on information theory. The first measure (*d*') describes the degree of interaction specialization at the species level, while the second measure (*H*_2_') characterizes the degree of specialization or partitioning among two parties in the entire network. Both indices are mathematically related and derived from Shannon entropy. The species-level index *d*' can be used to analyze variation within networks, while *H*_2_' as a network-level index is useful for comparisons across different interaction webs. Analyses of two published pollinator networks identified differences and features that have not been detected with previous approaches. For instance, plants and pollinators within a network differed in their average degree of specialization (weighted mean *d*'), and the correlation between specialization of pollinators and their relative abundance also differed between the webs. Rarefied sampling effort in both networks and null model simulations suggest that *H*_2_' is not affected by network size or sampling intensity.

**Conclusion:**

Quantitative analyses reflect properties of interaction networks more appropriately than previous qualitative attempts, and are robust against variation in sampling intensity, network size and symmetry. These measures will improve our understanding of patterns of specialization within and across networks from a broad spectrum of biological interactions.

## Background

The degree of specialization of plants or animals has been studied and debated extensively, and a continuum from complete specialization to full generalization can be found in various systems [1-6]. In general, two levels of specialization measures may be distinguished: first, the characterization of focal species and, second, the degree of specialization of an entire interaction network, representing an assemblage of species and their interaction partners (e.g. food webs, mutualistic networks, predator-prey relationships). When interactions are considered as ecological niche, the first level describes the niche breadth of a species and the second level the degree of niche partitioning across species. While the species level is more straightforward in its biological interpretation, analyses at the network level can be useful for comparisons across different types of networks. Such analyses have been performed to compare plant-pollinator webs versus plant-seed disperser webs [4,5], different plant-pollinator networks along geographic gradients [1,7,8], or food webs of variable size [9,10]. Entire network analyses are also used to study patterns on a community level such as coevolutionary adaptations [3], ecosystem stability or resilience [11-14].

### Quantifying specialization at the species level

Specialization or generalization of interactions are most commonly characterized as the number of partners (or 'links'), e.g. the number of pollinator species visiting a flowering plant species or the number of food plant families a herbivore feeds upon. In this qualitative approach, interactions between a consumer and a resource species are only scored in a binary way as 'present' or 'absent', ignoring any distinction between strong interactions and weak or occasional ones. For example, binary representation of interactions do not distinguish a scenario where 99% of the individuals of a herbivore species feed on a single plant species only, but occasionally an individual is found on another plant, from a different scenario where a herbivore regularly feeds on both food plants. The problem is analogous to the measurement of biodiversity either as a crude species richness versus as a more elaborate diversity index including relative abundances [15]. Several approaches have thus been used to directly include variation in interaction frequencies (i.e., their evenness) in characterizing the diversity of partners, e.g. Simpson's diversity index for pollinators [16,17] or Lloyd's index for host specificity [18]. Alternatively, other studies indirectly controlled for abundance or sampling intensity using rarefaction methods [13,19]. Correspondingly, Bersier and coworkers [20] have suggested to quantify the diversity of biomass flows in food webs using a Shannon diversity measure. Niche breadth theory provides several additional indices that include some measure of resource frequency or resource use intensity [21], which can be viewed in analogy to 'partner diversity' in the context of association networks. However, Hurlbert [22] emphasized that not only proportional utilization, but also the proportional availability of each niche should be taken into account. A species that uses all niches in the same proportion as their availability in the environment should be considered more opportunistic than a species that uses rare resources disproportionately more. If variation in resource availability is large, diversity-based measures that ignore this availability may be highly misleading [22,23]. Several niche breadth measures thus combine proportional resource utilization with proportional resource availability [22-24]. These concepts have been rarely applied in the context of species interaction networks, e.g. plant-pollinator webs where binary data are more common than quantitative webs.

### Quantifying specialization at the community level

The measurement used most commonly to characterize community-wide specialization is the 'connectance' index (*C*) [1,4,8-10,25-27]. *C *is defined as the proportion of the actually observed interactions to all possible interactions. Consider a contingency table showing the association between two parties, with *r *rows (e.g., plant species) and *c *columns (e.g., pollinators). Connectance is defined as *C *= *I*/(*r*·*c*), with *I *being the total number of non-zero elements in the matrix. Therefore, like the number of partners or links (*L*) described above, *C *uses only binary information and ignores interaction strength. *C *is directly related to the mean number of links (L¯
 MathType@MTEF@5@5@+=feaafiart1ev1aaatCvAUfKttLearuWrP9MDH5MBPbIqV92AaeXatLxBI9gBaebbnrfifHhDYfgasaacH8akY=wiFfYdH8Gipec8Eeeu0xXdbba9frFj0=OqFfea0dXdd9vqai=hGuQ8kuc9pgc9s8qqaq=dirpe0xb9q8qiLsFr0=vr0=vr0dc8meaabaqaciaacaGaaeqabaqabeGadaaakeaacuWGmbatgaqeaaaa@2DE5@) of plant species or pollinator species as *C *= L¯
 MathType@MTEF@5@5@+=feaafiart1ev1aaatCvAUfKttLearuWrP9MDH5MBPbIqV92AaeXatLxBI9gBaebbnrfifHhDYfgasaacH8akY=wiFfYdH8Gipec8Eeeu0xXdbba9frFj0=OqFfea0dXdd9vqai=hGuQ8kuc9pgc9s8qqaq=dirpe0xb9q8qiLsFr0=vr0=vr0dc8meaabaqaciaacaGaaeqabaqabeGadaaakeaacuWGmbatgaqeaaaa@2DE5@_*plants*_/*c *= L¯
 MathType@MTEF@5@5@+=feaafiart1ev1aaatCvAUfKttLearuWrP9MDH5MBPbIqV92AaeXatLxBI9gBaebbnrfifHhDYfgasaacH8akY=wiFfYdH8Gipec8Eeeu0xXdbba9frFj0=OqFfea0dXdd9vqai=hGuQ8kuc9pgc9s8qqaq=dirpe0xb9q8qiLsFr0=vr0=vr0dc8meaabaqaciaacaGaaeqabaqabeGadaaakeaacuWGmbatgaqeaaaa@2DE5@_*poll*_/*r*.

This measure, L¯
 MathType@MTEF@5@5@+=feaafiart1ev1aaatCvAUfKttLearuWrP9MDH5MBPbIqV92AaeXatLxBI9gBaebbnrfifHhDYfgasaacH8akY=wiFfYdH8Gipec8Eeeu0xXdbba9frFj0=OqFfea0dXdd9vqai=hGuQ8kuc9pgc9s8qqaq=dirpe0xb9q8qiLsFr0=vr0=vr0dc8meaabaqaciaacaGaaeqabaqabeGadaaakeaacuWGmbatgaqeaaaa@2DE5@, has also been used to compare networks [1,3,7,8,28]. Recently, it has been suggested to use L¯
 MathType@MTEF@5@5@+=feaafiart1ev1aaatCvAUfKttLearuWrP9MDH5MBPbIqV92AaeXatLxBI9gBaebbnrfifHhDYfgasaacH8akY=wiFfYdH8Gipec8Eeeu0xXdbba9frFj0=OqFfea0dXdd9vqai=hGuQ8kuc9pgc9s8qqaq=dirpe0xb9q8qiLsFr0=vr0=vr0dc8meaabaqaciaacaGaaeqabaqabeGadaaakeaacuWGmbatgaqeaaaa@2DE5@ instead of *C *to characterize networks [29]. However, note that comparisons across networks of different size (number of species) are problematic, since L¯
 MathType@MTEF@5@5@+=feaafiart1ev1aaatCvAUfKttLearuWrP9MDH5MBPbIqV92AaeXatLxBI9gBaebbnrfifHhDYfgasaacH8akY=wiFfYdH8Gipec8Eeeu0xXdbba9frFj0=OqFfea0dXdd9vqai=hGuQ8kuc9pgc9s8qqaq=dirpe0xb9q8qiLsFr0=vr0=vr0dc8meaabaqaciaacaGaaeqabaqabeGadaaakeaacuWGmbatgaqeaaaa@2DE5@, unlike *C*, is not scaled according to the number of available partners (see also [2,10]). L¯
 MathType@MTEF@5@5@+=feaafiart1ev1aaatCvAUfKttLearuWrP9MDH5MBPbIqV92AaeXatLxBI9gBaebbnrfifHhDYfgasaacH8akY=wiFfYdH8Gipec8Eeeu0xXdbba9frFj0=OqFfea0dXdd9vqai=hGuQ8kuc9pgc9s8qqaq=dirpe0xb9q8qiLsFr0=vr0=vr0dc8meaabaqaciaacaGaaeqabaqabeGadaaakeaacuWGmbatgaqeaaaa@2DE5@ in a small network may represent a larger proportion of available partners compared to the same value of L¯
 MathType@MTEF@5@5@+=feaafiart1ev1aaatCvAUfKttLearuWrP9MDH5MBPbIqV92AaeXatLxBI9gBaebbnrfifHhDYfgasaacH8akY=wiFfYdH8Gipec8Eeeu0xXdbba9frFj0=OqFfea0dXdd9vqai=hGuQ8kuc9pgc9s8qqaq=dirpe0xb9q8qiLsFr0=vr0=vr0dc8meaabaqaciaacaGaaeqabaqabeGadaaakeaacuWGmbatgaqeaaaa@2DE5@ in a large network.

Analyses based on binary data – both at the species and the community level – have obvious shortcomings, since they are highly dependent on sampling effort, decisions which species to include or not, and the size of investigated networks. Several authors thus emphasized the need to move beyond binary representations of interactions to quantitative measures involving some measure of interaction strength [4,20,27,29-32]. A way to at least partly overcome these deficiencies is to cut off all rare species or weak interactions below a frequency threshold [3,9,33,34] or to control for sampling effort in null models [7,8,13,19,25,35]. However, for interaction webs where a more detailed information is available, simplification to binary data as in *C *or L¯
 MathType@MTEF@5@5@+=feaafiart1ev1aaatCvAUfKttLearuWrP9MDH5MBPbIqV92AaeXatLxBI9gBaebbnrfifHhDYfgasaacH8akY=wiFfYdH8Gipec8Eeeu0xXdbba9frFj0=OqFfea0dXdd9vqai=hGuQ8kuc9pgc9s8qqaq=dirpe0xb9q8qiLsFr0=vr0=vr0dc8meaabaqaciaacaGaaeqabaqabeGadaaakeaacuWGmbatgaqeaaaa@2DE5@ remains unsatisfactory. Conveniently, the observed interaction frequency may represent a meaningful surrogate for interaction strength, at least in pollination and seed-dispersal systems as shown by Vázquez et al. [30] (see also [16]). Incorporating interaction frequency or even a direct measure of interaction strength in a network measure of specialization would thus provide an important progress frequently called for.

A severe additional problem of connectance is that its lower and upper constraints are not scale-invariant [25], which limits its use for comparisons across networks. The minimum possible value (*C*_min_) to maintain at least one link per species declines in a hyperbolic function with the number of interacting species, since *C*_min _= max(*r*, *c*)/(*r*·*c*), and an upper limit (*C*_max_) may be constrained by, or a function of, total sampling effort. Across networks, *C *decays strongly with network size, which has been debated in detail in the context of food web analysis [9,10,26,27,36,37]. The strong relationship between *C *and network size generates a problem for disentangling any biologically meaningful effect from this mathematically inherent scale dependence. For instance, network comparisons may focus on residual variation in *C *after an average effect of network size has been controlled for [1,4], or *C *could be rescaled to account for this size effect (see [25,36]). For natural networks of similar size, the range of actual *C *values is typically very narrow [4], thus other structural forces may be poorly detectable.

The objective of this paper is to develop and discuss specialization measures that are based on frequency data and thus account for sampling intensity, and that overcome the problem of scale dependence. We then test these approaches by evaluating the effect of sampling effort and scale dependence on a published natural pollination network, and on randomly generated associations as a null model. We differentiate between species-level measures of specialization, useful to investigate variability among species within a web, and a single network-wide measure that can be used for comparisons across networks.

## Results

### Patterns in two pollinator networks

Two selected plant-pollinator networks (British meadows studied by Memmott [32], Argentinean forests studied by Vázquez and Simberloff [33]) differ markedly in their degree of specialization when quantitative analyses are applied. The qualitative network index, connectance, is similar in both interaction webs (British web: *C *= 0.15, Argentinean web: *C *= 0.13). However, frequencies of pollinator visits are much more evenly distributed in the British community than in the Argentinean example. In the British web, the interaction between a dipteran species and *Leontodon hispidus *was the most frequent one, representing 6% of the total 2183 interactions observed. In the Argentinean network, visits of *Aristotelia chilensis *by a colletid bee species represented 20% of the 5285 interactions alone. Interactions between the top five plant and top five pollinator species made up 44% of the interactions in the British web, but 74% in the Argentinean web. This difference in the heterogeneity of interaction frequencies is not evident in measures based on binary information such as number of links (*L*) or connectance (*C*). In contrast, the degree of specialization shown by the frequency-based index *H*_2_' (standardized two-dimensional Shannon entropy, see *Methods: Network-level index*) is much lower in the British community (*H*_2_' = 0.24) compared to the Argentinean community (*H*_2_' = 0.63).

**Figure 1:**
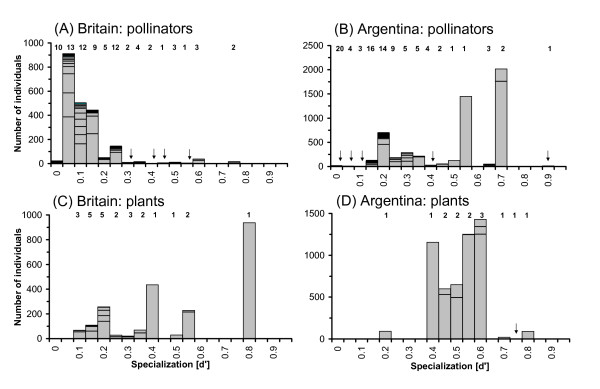
**Patterns within pollinator networks**. Frequency distribution of the species-level specialization index (*d*') for pollinators and plants from two published networks, one from Britain [32] and one from Argentina [33]. Bars show the number of individuals in each category (label '0' defines 0.00 ≤ *d*' < 0.05, etc.). Bars are separated for different species, and total number of species in each category is given on top. Arrows indicate cases where bars are invisible due to low numbers of individuals.

The variation of species-level specialization measures (standardized Kullback-Leibler distance, *d*') holds valuable information for the structural properties of a network (see *Methods: Species-level index*). The British pollination web is dominated by highly generalized pollinators (low *d*', both in terms of individuals as well as species), while putative specialists are represented by very few individuals and species (Fig. 1A). In contrast, most pollinators in the Argentinean web are moderately generalized to specialized, with the second highest level of specialization found in the most common species (Fig. 1B). Consequently, the weighted mean degree of specialization is much lower in the former web (<*d*'_*poll*_> = 0.16) than in the latter (<*d*'_*poll*_> = 0.54). The relationship between specialization of species *i *(*d*'*_i_*) and its interaction frequency (*A*_*i*_) across the pollinator species differs between the two webs. In the British web, *d*'_*i *_and *A*_*i *_were not correlated significantly (Spearman's *r*_*s *_= -0.08, *p *= 0.46), while a highly positive correlation was found in the Argentinean web (*r*_*s *_= 0.65, *p *< 0.0001). Note that designation of any specialization index to a species *i *that is only represented by a single individual may be critical. However, significances in the above correlations remain unaffected when pollinators with one single interaction are excluded. From the plants' point of view, the species in Memmott's web are also more generalized in terms of their pollinator spectrum (Fig. 1C) than the plants studied by Vázquez and Simberloff (Fig. 1D). The respective weighted means are <*d*'_*plants*_> = 0.27 and <*d*'_*plants*_> = 0.53. No significant correlation was found between the plants' frequency and specialization in either web (both *p *≥ 0.16). Interestingly, plants were on average more specialized than pollinators in the British web (<*d*'_*plants*_> > <*d*'_*poll.*_>), but not in the Argentinean web. This distinction is not found when only the weighted mean number of links (*L*) are examined, since <*L*_*plants*_> is much greater than <*L*_*poll.*_> in both networks. The difference in <*L*> may be driven by the highly asymmetrical matrix architecture in both webs, where the number of pollinator species greatly exceeds the number of plant species. The unweighted mean L¯
 MathType@MTEF@5@5@+=feaafiart1ev1aaatCvAUfKttLearuWrP9MDH5MBPbIqV92AaeXatLxBI9gBaebbnrfifHhDYfgasaacH8akY=wiFfYdH8Gipec8Eeeu0xXdbba9frFj0=OqFfea0dXdd9vqai=hGuQ8kuc9pgc9s8qqaq=dirpe0xb9q8qiLsFr0=vr0=vr0dc8meaabaqaciaacaGaaeqabaqabeGadaaakeaacuWGmbatgaqeaaaa@2DE5@ is even directly linked to the matrix architecture (i.e., number of rows and columns, *r *and *c*) by a constant (connectance *C*), since L¯
 MathType@MTEF@5@5@+=feaafiart1ev1aaatCvAUfKttLearuWrP9MDH5MBPbIqV92AaeXatLxBI9gBaebbnrfifHhDYfgasaacH8akY=wiFfYdH8Gipec8Eeeu0xXdbba9frFj0=OqFfea0dXdd9vqai=hGuQ8kuc9pgc9s8qqaq=dirpe0xb9q8qiLsFr0=vr0=vr0dc8meaabaqaciaacaGaaeqabaqabeGadaaakeaacuWGmbatgaqeaaaa@2DE5@_*r *_= *c*·*C *and L¯
 MathType@MTEF@5@5@+=feaafiart1ev1aaatCvAUfKttLearuWrP9MDH5MBPbIqV92AaeXatLxBI9gBaebbnrfifHhDYfgasaacH8akY=wiFfYdH8Gipec8Eeeu0xXdbba9frFj0=OqFfea0dXdd9vqai=hGuQ8kuc9pgc9s8qqaq=dirpe0xb9q8qiLsFr0=vr0=vr0dc8meaabaqaciaacaGaaeqabaqabeGadaaakeaacuWGmbatgaqeaaaa@2DE5@_*c *_= *r*·*C*. In contrast, the matrix asymmetry does not affect *d*' (see also below, *Null model patterns*).

### Simulation of sampling effort

**Figure 2:**
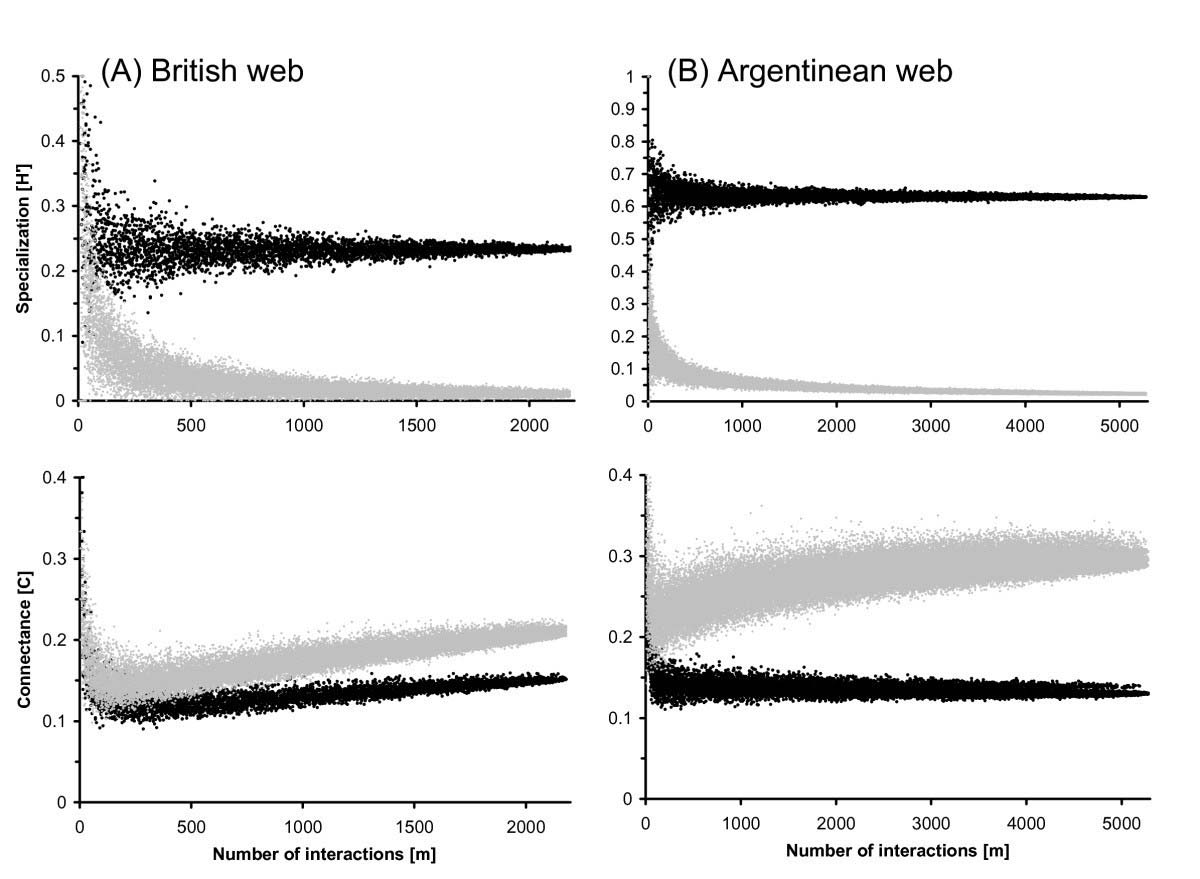
**Sampling effect in pollinator networks**. Rarefaction of sampling effort in a British and an Argentinean pollination web [32,33]. Two network-level measures of specialization – the frequency-based specialization index (*H*_2_') and the 'connectance' index (*C*) – are shown for networks in which the total number of interactions (*m*) has been reduced by randomly deleting interactions. Black dots show the effect of sampling effort for the original association matrix, gray dots the effect for a null model, i.e. five networks in which partners were randomly associated (same row and column totals as in the original matrix).

In order to test whether specialization estimates are dependent on sampling and scale effects, we simulated a decreased sampling intensity in both networks using rarefaction (see *Methods: Simulation of sampling effort and matrix architecture*). In both networks, *H*_2_' is robust and already very well estimated by a small fraction of the interactions sampled (Fig. 2). The coefficient of variance of *H*_2_' remains below 5% from about half of the total number of visits onwards in the British web and even at one-tenth of the total sampling effort of the Argentinean web. The estimation of connectance (*C*) is also relatively stable at least in the Argentinean web, although it shows a positive trend across sampling effort in the British web (Fig. 2). These findings suggest that network-wide measures of specialization, particularly *H*_2_', do not necessarily require a very large or even complete association matrix, but can also be very well estimated from a smaller representative subset as long as there is no systematic sampling bias.

### Null model patterns

The degree of specialization can be further characterized by comparison with a null model. The null model used here is that each species has a fixed total number of interactions (given by the observed association matrix), but interactions are assigned randomly. In the above pollinator networks, random associations yield a specialization index *H*_2_' that remains close to zero for almost the entire range of sampling intensity, while connectance (*C*) shows a positive trend over the total number of interactions (*m*) (Fig. 2). Therefore, *H*_2_' derived from real networks may typically be clearly distinguished from this null model, while the comparison of *C *is complicated by scale dependence and the relatively large values yielded by the null model.

**Figure 3:**
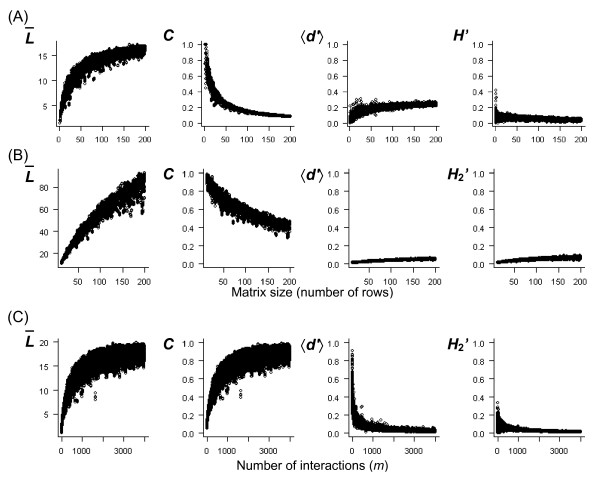
**Simulated random networks**. Behavior of specialization measures in simulated random networks. Each point represents one matrix with random associations, based on specific row and column totals that follow a lognormal distribution. The size of squared matrices in (A) increased from 2 × 2 to 200 × 200. In (B), only the number of rows changed, while the number of columns was fixed at 20, rectangular matrices thus increased from 2 × 20 to 200 × 20. In (C), the network size was fixed at 20 × 20. The total number of interactions (*m*) increased with matrix size in (A), where each species had on average 20 individuals. In (B), *m *was fixed at 4000, resulting in a reduced interaction density for larger matrices. In (C), *m *increased from 20 to 4000. The index *H*_2_' and connectance *C *are specialization measures of the whole matrix and thus reciprocal, while the average number of links (L¯
 MathType@MTEF@5@5@+=feaafiart1ev1aaatCvAUfKttLearuWrP9MDH5MBPbIqV92AaeXatLxBI9gBaebbnrfifHhDYfgasaacH8akY=wiFfYdH8Gipec8Eeeu0xXdbba9frFj0=OqFfea0dXdd9vqai=hGuQ8kuc9pgc9s8qqaq=dirpe0xb9q8qiLsFr0=vr0=vr0dc8meaabaqaciaacaGaaeqabaqabeGadaaakeaacuWGmbatgaqeaaaa@2DE5@), and weighted mean standardized Kullback-Leibler distance (<*d*'>) are given for all columns (rows give a similar pattern).

Simulations of artificially generated random associations (see *Methods: Simulation of sampling effort and matrix architecture*) confirm that the network-level specialization index *H*_2_' is largely unaffected by network size (Fig. 3A), network architecture (Fig. 3B) or total number of interactions (*m*) for a fixed matrix size (Fig. 3C). For random associations as shown here, *H*_2_' is usually close to zero. Connectance values (*C*) of random matrices show the known hyperbolic function over the number of associated species (Fig. 3A), changes with matrix asymmetry (Fig. 3B) and increase strongly with increasing *m *(Fig. 3C). For specialization measures at the species level, the average number of links per species (L¯
 MathType@MTEF@5@5@+=feaafiart1ev1aaatCvAUfKttLearuWrP9MDH5MBPbIqV92AaeXatLxBI9gBaebbnrfifHhDYfgasaacH8akY=wiFfYdH8Gipec8Eeeu0xXdbba9frFj0=OqFfea0dXdd9vqai=hGuQ8kuc9pgc9s8qqaq=dirpe0xb9q8qiLsFr0=vr0=vr0dc8meaabaqaciaacaGaaeqabaqabeGadaaakeaacuWGmbatgaqeaaaa@2DE5@) increases strongly with network size, number of available partners, and *m *(Fig. 3). While other niche breadth measures may also show some variation across different network scales (not shown), the weighted mean Kullback-Leibler distance <*d*'> is poorly affected by network size, network asymmetry, and number of interactions (Fig. 3). Both *H*_2_' and *d*' may thus be appropriate for comparisons across matrices of different scale.

## Discussion

### Properties of specialization measures

The suggested indices, *d*' and *H*_2_', quantify the degree of specialisation of elements within an interaction network and of the entire network, respectively. While the number of links (*L*) and connectance (*C*) represent species-level and community-level measures of interactions based on binary data, respectively, *d*' and *H*_2_' represent corresponding measures for frequency-based data. The need to include information on interaction strength or interaction frequency into network analyses has been announced by various authors [4,20,27,30,31,38]. Parallel to earlier advances in diversity measures compared to species richness, quantitative network measures account for the heterogeneity in link strength rather than assigning equal weights to every link. Moreover, we have shown that *d*' and *H*_2_' are largely robust against variation in matrix size, shape, and sampling effort. In several cases, *C *may be strongly affected by sampling effort [25,27], while *H*_2_' remained largely unchanged in simulations of random associations over a range of network sizes, variable network asymmetries, and number of interactions. This scale invariance suggests that both *d*' and *H*_2_' can be used directly for comparisons across different networks, while comparisons of *L *and *C *are more problematic [1,35].

Qualitative methods like the indices suggested here also allow a more detailed analysis of interaction patterns within and across networks. Fruitful areas include comparisons of networks across different interaction types [4], biogeographical gradients [1], biodiversity and land use gradients [13], robustness of networks against extinction risks [39], asymmetries between plants and animals [38], and relationships between specialisation and abundance [35]. While a comparison of the average number of partners between plants versus animals is solely dependent on the matrix architecture (*i.e*., the number of rows *r *versus columns *c*, since L¯
 MathType@MTEF@5@5@+=feaafiart1ev1aaatCvAUfKttLearuWrP9MDH5MBPbIqV92AaeXatLxBI9gBaebbnrfifHhDYfgasaacH8akY=wiFfYdH8Gipec8Eeeu0xXdbba9frFj0=OqFfea0dXdd9vqai=hGuQ8kuc9pgc9s8qqaq=dirpe0xb9q8qiLsFr0=vr0=vr0dc8meaabaqaciaacaGaaeqabaqabeGadaaakeaacuWGmbatgaqeaaaa@2DE5@_*plants *_= *c*·*C *and L¯
 MathType@MTEF@5@5@+=feaafiart1ev1aaatCvAUfKttLearuWrP9MDH5MBPbIqV92AaeXatLxBI9gBaebbnrfifHhDYfgasaacH8akY=wiFfYdH8Gipec8Eeeu0xXdbba9frFj0=OqFfea0dXdd9vqai=hGuQ8kuc9pgc9s8qqaq=dirpe0xb9q8qiLsFr0=vr0=vr0dc8meaabaqaciaacaGaaeqabaqabeGadaaakeaacuWGmbatgaqeaaaa@2DE5@_*poll *_= *r*·*C*), this limitation does not apply to *d*'. In the two selected pollinator webs, plants are either similarly or more specialised than pollinators in regard to weighted mean *d*'. This allows an scale-independent evaluation of asymmetries in the degree of specialization between partners (see also [38]). Moreover, Vázquez and Aizen [35] noted that the number of links of a species (*L*_*i*_) is strongly positively correlated with its overall frequency (*A*_*i*_) in five pollination networks including the datasets analyzed above. They argued that this apparent higher generalization of common plants and common pollinators may be largely explained by null models, calling for an improved measurement of specialization. Our results for the correlation between *d*'_*i *_and *A*_*i *_in two pollinator webs suggest that the relationship between specialization and abundance may be more variable, and even positive as in the Argentinean network.

### Caveats

Some problems apply to any measure of network analyses including the proposed indices. Measures of specialization mostly ignore phylogenetic relationships or ecological similarity within an association matrix. For example, a plant species that is pollinated by multiple moth species may be unsuitably regarded as more generalized than a plant pollinated by few insect species comprising several different orders [40]. In addition, the fact that herbivores are commonly specialized on host plant families rather than species may skew network patterns if not carefully accounted for. A first approach to investigate such effects may be to compare the level of specialization after a stepwise reduction of the matrix by pooling species to higher taxonomic units, such as genera, families, and orders. For known phylogenies, more advanced techniques for analyses with a particular evolutionary focus are available [41-43]. Another deficiency may be that species or their partners are all given the same individual 'weight' in the analyses, whether they may be small bees or large bats visiting a small herb with little nectar or a mass flowering tree. Null models as in the calculation for both *C *and *H*_2_.' imply that all individuals can be shifted around between resources in the same way, irrespective of their size or non-fitting parameters. The role of 'forbidden links' as constraints to network analyses has been discussed elsewhere [44,45]. Similarly, calculations of *d*' or other niche breadth measures are based on the implicit assumption that each species adjusts its interactions according to the availability of partners (niches), irrespective of morphological or behavioral constraints. Moreover, if data are collected from a large heterogeneous habitat or over a prolonged time period, calculations of the degree of specialization may be severely constrained by the spatiotemporal overlap or non-overlap between partners for other reasons than resource preferences, e.g. when not all species are able to reach all sites in the same way, or when some resources and consumers have asynchronous phenologies. Consequently, network analyses as suggested here will be most useful to study resource-consumer partitioning within a short time frame and limited spatial scale.

For both indices *d*' and *H*_2_', we proposed above to use the total number of interactions for each species as a measure of partner availability (*q*_*j*_) and as constraint for standardization (fixed row and column totals). It may be debated whether independent measures of plant and animal abundances could be more appropriate than using interaction frequency data as such. However, despite the fact that such abundance data barely exist for most networks, note that the actual number of interactions often more suitably reflects resource availability and consumer activity than an independent measure of species abundance. For instance, a flower of one species may have a much higher nectar production than another and consequently receive a higher number of visitors, while the local abundance of the plant species does not reflect such differences in resource quality and/or quantity. Both *d*' and *H*_2_' thus focus on the actual partitioning between the interacting species. In studies where detailed knowledge or theoretical assumptions about resources (availability and quality) or consumers (activity density and consumption rate) are available or under experimental control, such data may be incorporated into the analysis (defining *q*_*j *_and constraints) instead of interaction frequencies. The constraint of fixed row and column totals has been debated elsewhere in the context of species co-occurrence patterns, where it was found to be most appropriate in null model comparisons, although critics have argued earlier that these marginals themselves may already reflect competitive interactions ([46] and references therein). Any approach to compare networks based on fixed marginals for standardization will fail to detect potentially meaningful patterns displayed by these architectural features, namely the number of resource and consumer species and the heterogeneity of total interaction frequencies. This network architecture may already be shaped by past competitive interactions or indicate fundamental constraints, a largely unexplored hypothesis that merits additional investigations.

It should also be emphasized that analyses of frequency data may be susceptible for pseudoreplication of repeated associations of the same individuals or close associations derived from a single dispersal event (e.g. a social insect colony, aggregating individuals, multiple offspring from a single egg cluster, or monospecific plant clusters). These may lead to an overestimation of specialization. To be more meaningful on a population level, frequency analyses should thus be based on spatially independent association replicates. Note that all species-wise specialization measures such as *d*' are sensitive to the behavior of the other species. Any systematic sampling bias (e.g. a taxonomic focus within a guild) will therefore affect the conclusions of comparisons within or across networks.

## Conclusion

In accordance with previous calls [4,20,27,30,31,38], we suggest that the explicit inclusion of frequency data reflects an important step forward in network analyses, as too many assumptions are implicit in any measure based on binary representation. Most notably, connectance and 'number of partners' imply an equal availability of all partners – an unlikely scenario. Qualitative indices are not robust against sampling effort. On the contrary, the proposed quantitative measures based on interaction frequencies explicitly account for this source of variation. Our study suggests that *d*' and *H*_2_' represent scale-independent and meaningful indices to characterize specialization on the level of single species and the entire network, respectively. These novel indices allow us to investigate patterns within and across networks that have not been detected with qualitative measures such as correlations with species frequencies, network size and asymmetries in specialization between partners. Recently, Bascompte et al. [38] showed that the incorporation of frequency data may unveil pervasive asymmetries within networks. Particularly since Vázquez et al. [30] demonstrated that interaction frequencies in plant-pollinator and plant-seed disperser systems often correlate with the magnitude of mutualistic services for the plant (although variation in pollinator effectiveness can be important, see [47]), an increased collection of frequency data and appropriate quantitative analyses would greatly benefit future network studies.

## Methods

### Species-level index

As species-level measure of 'partner diversity', we propose the Kullback-Leibler distance (or Kullback-Leibler divergence, relative entropy) in a standardized form (*d*'). Coming from information theory, this index quantifies the difference between two probability distributions [48]. While the standardized Hurlbert's and Smith's measure of niche breadth could be used alternatively [21,22,24], *d*' has some advantages in the context of networks. While all three indices regard an exclusive pairing between two species as high degree of specialization as long as interactions between the two partners are infrequent, Hurlbert's and Smith's indices show a undesired trend towards full generalization when the number of interactions between the two partners increase, although this should be considered a stronger indication of specialization (see below, *Properties of alternative niche breadth measures*). The interaction between two parties is commonly displayed in a *r *× *c *contingency table, with *r *rows representing one party such as flowering plant species, and *c *columns representing the other party such as pollinator species. In each cell, the frequency of interaction between plant species *i *and pollinator species *j *(or another useful measure of interaction strength) is given as *a*_*ij*_, (Table 1).

Instead of frequencies (*a*_*ij*_), each interaction can be assigned a proportion of the total (*m*) as

pij=aij/m
 MathType@MTEF@5@5@+=feaafiart1ev1aaatCvAUfKttLearuWrP9MDH5MBPbIqV92AaeXatLxBI9gBaebbnrfifHhDYfgasaacH8akY=wiFfYdH8Gipec8Eeeu0xXdbba9frFj0=OqFfea0dXdd9vqai=hGuQ8kuc9pgc9s8qqaq=dirpe0xb9q8qiLsFr0=vr0=vr0dc8meaabaqaciaacaGaaeqabaqabeGadaaakeaacqWGWbaCdaWgaaWcbaGaemyAaKMaemOAaOgabeaakiabg2da9iabdggaHnaaBaaaleaacqWGPbqAcqWGQbGAaeqaaOGaei4la8IaemyBa0gaaa@388B@, where ∑i=1r∑j=1cpij=1
 MathType@MTEF@5@5@+=feaafiart1ev1aaatCvAUfKttLearuWrP9MDH5MBPbIqV92AaeXatLxBI9gBaebbnrfifHhDYfgasaacH8akY=wiFfYdH8Gipec8Eeeu0xXdbba9frFj0=OqFfea0dXdd9vqai=hGuQ8kuc9pgc9s8qqaq=dirpe0xb9q8qiLsFr0=vr0=vr0dc8meaabaqaciaacaGaaeqabaqabeGadaaakeaadaaeWbqaamaaqahabaGaemiCaa3aaSbaaSqaaiabdMgaPjabdQgaQbqabaaabaGaemOAaOMaeyypa0JaeGymaedabaGaem4yamganiabggHiLdaaleaacqWGPbqAcqGH9aqpcqaIXaqmaeaacqWGYbGCa0GaeyyeIuoakiabg2da9iabigdaXaaa@40D2@.

Let *p*'_*ij *_be the proportion of the number of interactions (*a*_*ij*_) in relation to the respective row total (*A*_*i*_), and *q*_*j *_the proportion of all interactions by partner *j *in relation to the total number of interactions (*m*). Thus,

p′ij=aij/Ai
 MathType@MTEF@5@5@+=feaafiart1ev1aaatCvAUfKttLearuWrP9MDH5MBPbIqV92AaeXatLxBI9gBaebbnrfifHhDYfgasaacH8akY=wiFfYdH8Gipec8Eeeu0xXdbba9frFj0=OqFfea0dXdd9vqai=hGuQ8kuc9pgc9s8qqaq=dirpe0xb9q8qiLsFr0=vr0=vr0dc8meaabaqaciaacaGaaeqabaqabeGadaaakeaacuWGWbaCgaqbamaaBaaaleaacqWGPbqAcqWGQbGAaeqaaOGaeyypa0Jaemyyae2aaSbaaSqaaiabdMgaPjabdQgaQbqabaGccqGGVaWlcqWGbbqqdaWgaaWcbaGaemyAaKgabeaaaaa@39C6@, ∑j=1cp′ij=1
 MathType@MTEF@5@5@+=feaafiart1ev1aaatCvAUfKttLearuWrP9MDH5MBPbIqV92AaeXatLxBI9gBaebbnrfifHhDYfgasaacH8akY=wiFfYdH8Gipec8Eeeu0xXdbba9frFj0=OqFfea0dXdd9vqai=hGuQ8kuc9pgc9s8qqaq=dirpe0xb9q8qiLsFr0=vr0=vr0dc8meaabaqaciaacaGaaeqabaqabeGadaaakeaadaaeWbqaaiqbdchaWzaafaWaaSbaaSqaaiabdMgaPjabdQgaQbqabaaabaGaemOAaOMaeyypa0JaeGymaedabaGaem4yamganiabggHiLdGccqGH9aqpcqaIXaqmaaa@39DE@, qj=Aj/m
 MathType@MTEF@5@5@+=feaafiart1ev1aaatCvAUfKttLearuWrP9MDH5MBPbIqV92AaeXatLxBI9gBaebbnrfifHhDYfgasaacH8akY=wiFfYdH8Gipec8Eeeu0xXdbba9frFj0=OqFfea0dXdd9vqai=hGuQ8kuc9pgc9s8qqaq=dirpe0xb9q8qiLsFr0=vr0=vr0dc8meaabaqaciaacaGaaeqabaqabeGadaaakeaacqWGXbqCdaWgaaWcbaGaemOAaOgabeaakiabg2da9iabdgeabnaaBaaaleaacqWGQbGAaeqaaOGaei4la8IaemyBa0gaaa@3597@, and ∑j=1cqj=1
 MathType@MTEF@5@5@+=feaafiart1ev1aaatCvAUfKttLearuWrP9MDH5MBPbIqV92AaeXatLxBI9gBaebbnrfifHhDYfgasaacH8akY=wiFfYdH8Gipec8Eeeu0xXdbba9frFj0=OqFfea0dXdd9vqai=hGuQ8kuc9pgc9s8qqaq=dirpe0xb9q8qiLsFr0=vr0=vr0dc8meaabaqaciaacaGaaeqabaqabeGadaaakeaadaaeWbqaaiabdghaXnaaBaaaleaacqWGQbGAaeqaaaqaaiabdQgaQjabg2da9iabigdaXaqaaiabdogaJbqdcqGHris5aOGaeyypa0JaeGymaedaaa@3879@.

To quantify the specialization of a species *i*, the following index *d*_*i *_is suggested. This *d*_*i *_is related to Shannon diversity, similar to an index recently suggested to characterize biomass flow diversity in food webs [20]. However, an appropriate index in this context should not only consider the diversity of partners, but also their respective availability (see [22]). Consequently, the following index compares the distribution of the interactions with each partner (*p*'_*j*_) to the overall partner availability (*q*_*j*_). The Kullback-Leibler distance for species *i *is denoted as

di=∑j=1c(p′ij⋅ln⁡p′ijqj),
 MathType@MTEF@5@5@+=feaafiart1ev1aaatCvAUfKttLearuWrP9MDH5MBPbIqV92AaeXatLxBI9gBaebbnrfifHhDYfgasaacH8akY=wiFfYdH8Gipec8Eeeu0xXdbba9frFj0=OqFfea0dXdd9vqai=hGuQ8kuc9pgc9s8qqaq=dirpe0xb9q8qiLsFr0=vr0=vr0dc8meaabaqaciaacaGaaeqabaqabeGadaaakeaacqWGKbazdaWgaaWcbaGaemyAaKgabeaakiabg2da9maaqahabaWaaeWaceaacuWGWbaCgaqbamaaBaaaleaacqWGPbqAcqWGQbGAaeqaaOGaeyyXICTagiiBaWMaeiOBa42aaSaaaeaacuWGWbaCgaqbamaaBaaaleaacqWGPbqAcqWGQbGAaeqaaaGcbaGaemyCae3aaSbaaSqaaiabdQgaQbqabaaaaaGccaGLOaGaayzkaaaaleaacqWGQbGAcqGH9aqpcqaIXaqmaeaacqWGJbWya0GaeyyeIuoakiabcYcaSaaa@4AD1@

which can be normalized as

d′i=di−dmin⁡dmax⁡−dmin⁡.
 MathType@MTEF@5@5@+=feaafiart1ev1aaatCvAUfKttLearuWrP9MDH5MBPbIqV92AaeXatLxBI9gBaebbnrfifHhDYfgasaacH8akY=wiFfYdH8Gipec8Eeeu0xXdbba9frFj0=OqFfea0dXdd9vqai=hGuQ8kuc9pgc9s8qqaq=dirpe0xb9q8qiLsFr0=vr0=vr0dc8meaabaqaciaacaGaaeqabaqabeGadaaakeaacuWGKbazgaqbamaaBaaaleaacqWGPbqAaeqaaOGaeyypa0ZaaSaaaeaacqWGKbazdaWgaaWcbaGaemyAaKgabeaakiabgkHiTiabdsgaKnaaBaaaleaacyGGTbqBcqGGPbqAcqGGUbGBaeqaaaGcbaGaemizaq2aaSbaaSqaaiGbc2gaTjabcggaHjabcIha4bqabaGccqGHsislcqWGKbazdaWgaaWcbaGagiyBa0MaeiyAaKMaeiOBa4gabeaaaaGccqGGUaGlaaa@474F@

The theoretical maximum is given by *d*_max _= ln (*m*/*A*_*i*_), and the theoretical minimum (*d*_min_) is zero for the special case where all *p*'_*ij *_= *q*_*j*_. However, a realistic *d*_min _may be constrained at some value above zero given that *p*'_*ij *_and *q*_*j *_are calculated from discrete integer values (*a*_*ij*_). To take this into account, *d*_min _is more suitably computed algorithmically as in a program available from the authors and online [49], providing all *d*' for a given matrix. This standardized Kullback-Leibler distance (*d*') ranges from 0 for the most generalized to 1.0 for the most specialized case. Thus, *d*' can be interpreted as deviation of the actual interaction frequencies from a null model which assumes that all partners are used in proportion to their availability. An average degree of specialization among the species of a party can be presented as a weighted mean of the standardized index, e.g. <*d*'_*i*_> for pollinators as

〈d′i〉=1m∑i=1r(d′i⋅Ai)=∑i=1r(d′i⋅qi)
 MathType@MTEF@5@5@+=feaafiart1ev1aaatCvAUfKttLearuWrP9MDH5MBPbIqV92AaeXatLxBI9gBaebbnrfifHhDYfgasaacH8akY=wiFfYdH8Gipec8Eeeu0xXdbba9frFj0=OqFfea0dXdd9vqai=hGuQ8kuc9pgc9s8qqaq=dirpe0xb9q8qiLsFr0=vr0=vr0dc8meaabaqaciaacaGaaeqabaqabeGadaaakeaacqGHPms4cuWGKbazgaqbamaaBaaaleaacqWGPbqAaeqaaOGaeyOkJeVaeyypa0ZaaSaaaeaacqaIXaqmaeaacqWGTbqBaaWaaabCaeaadaqadiqaaiqbdsgaKzaafaWaaSbaaSqaaiabdMgaPbqabaGccqGHflY1cqWGbbqqdaWgaaWcbaGaemyAaKgabeaaaOGaayjkaiaawMcaaaWcbaGaemyAaKMaeyypa0JaeGymaedabaGaemOCaihaniabggHiLdGccqGH9aqpdaaeWbqaamaabmGabaGafmizaqMbauaadaWgaaWcbaGaemyAaKgabeaakiabgwSixlabdghaXnaaBaaaleaacqWGPbqAaeqaaaGccaGLOaGaayzkaaaaleaacqWGPbqAcqGH9aqpcqaIXaqmaeaacqWGYbGCa0GaeyyeIuoaaaa@58B4@

While <*d*'_*i*_> usually differs from <*d*'_*j*_>, the weighted means of the non-standardized Kullback-Leibler distances are the same for both parties, hence <*d*_*i*_> = <*d*_*j*_>.

### Network-level index

The following network-wide measure is based on the bipartite representation of a two mode network of interactions such as plant-animal or other resource-consumer interactions where members of each party interact with members of the other party but not among themselves (unlike many food webs). The two-dimensional Shannon entropy (termed *H*_2 _in order to avoid confusion with the common one-dimensional *H*) is obtained as

H2=−∑i=1r∑j=1c(pij⋅ln⁡pij).
 MathType@MTEF@5@5@+=feaafiart1ev1aaatCvAUfKttLearuWrP9MDH5MBPbIqV92AaeXatLxBI9gBaebbnrfifHhDYfgasaacH8akY=wiFfYdH8Gipec8Eeeu0xXdbba9frFj0=OqFfea0dXdd9vqai=hGuQ8kuc9pgc9s8qqaq=dirpe0xb9q8qiLsFr0=vr0=vr0dc8meaabaqaciaacaGaaeqabaqabeGadaaakeaacqWGibasdaWgaaWcbaGaeGOmaidabeaakiabg2da9iabgkHiTmaaqahabaWaaabCaeaadaqadiqaaiabdchaWnaaBaaaleaacqWGPbqAcqWGQbGAaeqaaOGaeyyXICTagiiBaWMaeiOBa4MaemiCaa3aaSbaaSqaaiabdMgaPjabdQgaQbqabaaakiaawIcacaGLPaaaaSqaaiabdQgaQjabg2da9iabigdaXaqaaiabdogaJbqdcqGHris5aaWcbaGaemyAaKMaeyypa0JaeGymaedabaGaemOCaihaniabggHiLdGccqGGUaGlaaa@4EFB@

*H*_2 _decreases with higher specialization. This measure is closely related to the weighted mean of the non-standardized Kullback-Leibler distance of all species, since

<*d*_*i*_> = <*d*_*j*_> = *H*_2max _- *H*_2_

(see below, *Relationship between d*_*i *_*and H*_2_). *H*_2 _can be standardized between 0 and 1.0 for extreme specialization versus extreme generalization, respectively, when its minimum and maximum values (*H*_2min _and *H*_2max_) are known. *H*_2min _and *H*_2max _can be calculated for given constraints. The constraints used here are the maintenance of the total number of interactions of each species, thus all row and column totals, *A*_*i *_and *A*_*j*_, being fixed (see also [46]). Alternative constraints may be defined depending on the knowledge of the system studied.

*H*_2 _reaches its theoretical maximum where each *p*_*ij *_equals its expected value from a random interaction matrix (*q*_*i*_·*q*_*j*_), such that

H2max⁡=−∑i=1r∑j=1c(qiqj⋅ln⁡qiqj),
 MathType@MTEF@5@5@+=feaafiart1ev1aaatCvAUfKttLearuWrP9MDH5MBPbIqV92AaeXatLxBI9gBaebbnrfifHhDYfgasaacH8akY=wiFfYdH8Gipec8Eeeu0xXdbba9frFj0=OqFfea0dXdd9vqai=hGuQ8kuc9pgc9s8qqaq=dirpe0xb9q8qiLsFr0=vr0=vr0dc8meaabaqaciaacaGaaeqabaqabeGadaaakeaacqWGibasdaWgaaWcbaGaeGOmaiJagiyBa0MaeiyyaeMaeiiEaGhabeaakiabg2da9iabgkHiTmaaqahabaWaaabCaeaadaqadiqaaiabdghaXnaaBaaaleaacqWGPbqAaeqaaOGaemyCae3aaSbaaSqaaiabdQgaQbqabaGccqGHflY1cyGGSbaBcqGGUbGBcqWGXbqCdaWgaaWcbaGaemyAaKgabeaakiabdghaXnaaBaaaleaacqWGQbGAaeqaaaGccaGLOaGaayzkaaaaleaacqWGQbGAcqGH9aqpcqaIXaqmaeaacqWGJbWya0GaeyyeIuoaaSqaaiabdMgaPjabg2da9iabigdaXaqaaiabdkhaYbqdcqGHris5aOGaeiilaWcaaa@5663@

while its theoretical minimum (*H*_2min_) may be close to zero depending on the matrix architecture. Like for *d*_min_ above, H_2max _and H_2min _are constrained by the fact that they are derived from integer values. A program implementing a heuristic solution to obtain *H*_2max _and *H*_2min_, and to perform the entire analysis is available from the authors or online [49].

The degree of specialization is obtained as a standardized entropy on a scale between *H*_2min _and *H*_2max _as

H′2=H2max⁡−H2H2max⁡−H2min⁡.
 MathType@MTEF@5@5@+=feaafiart1ev1aaatCvAUfKttLearuWrP9MDH5MBPbIqV92AaeXatLxBI9gBaebbnrfifHhDYfgasaacH8akY=wiFfYdH8Gipec8Eeeu0xXdbba9frFj0=OqFfea0dXdd9vqai=hGuQ8kuc9pgc9s8qqaq=dirpe0xb9q8qiLsFr0=vr0=vr0dc8meaabaqaciaacaGaaeqabaqabeGadaaakeaacuWGibasgaqbamaaBaaaleaacqaIYaGmaeqaaOGaeyypa0ZaaSaaaeaacqWGibasdaWgaaWcbaGaeGOmaiJagiyBa0MaeiyyaeMaeiiEaGhabeaakiabgkHiTiabdIeainaaBaaaleaacqaIYaGmaeqaaaGcbaGaemisaG0aaSbaaSqaaiabikdaYiGbc2gaTjabcggaHjabcIha4bqabaGccqGHsislcqWGibasdaWgaaWcbaGaeGOmaiJagiyBa0MaeiyAaKMaeiOBa4gabeaaaaGccqGGUaGlaaa@483F@

Consequently, *H*_2_' ranges between 0 and 1.0 for extreme generalization and specialization, respectively.

### Comparison with random associations

*H*_2 _can be tested against a null model of random associations (*H*_2ran_). A number of random permutations of the matrix can be performed using a *r *× *c *randomization algorithm (also available at [49]). The probability (*p*-value) that the observed *H*_2 _is more specialized than expected by random associations is simply given as the proportion of values obtained for *H*_2ran _that are equal or larger than *H*_2_, a common procedure in randomization statistics [25,50]. *H*_2ran _is usually only slightly larger than H_2min_.Previously, permutations of *r *× *c *contingency tables often used a different test statistics instead of *H*_2 _[25,51,52]:

T=−∑i=1r∑j=1c(aij.ln⁡aij).
 MathType@MTEF@5@5@+=feaafiart1ev1aaatCvAUfKttLearuWrP9MDH5MBPbIqV92AaeXatLxBI9gBaebbnrfifHhDYfgasaacH8akY=wiFfYdH8Gipec8Eeeu0xXdbba9frFj0=OqFfea0dXdd9vqai=hGuQ8kuc9pgc9s8qqaq=dirpe0xb9q8qiLsFr0=vr0=vr0dc8meaabaqaciaacaGaaeqabaqabeGadaaakeaacqWGubavcqGH9aqpcqGHsisldaaeWbqaamaaqahabaWaaeWaceaacqWGHbqydaWgaaWcbaGaemyAaKMaemOAaOgabeaakiabc6caUiGbcYgaSjabc6gaUjabdggaHnaaBaaaleaacqWGPbqAcqWGQbGAaeqaaaGccaGLOaGaayzkaaaaleaacqWGQbGAcqGH9aqpcqaIXaqmaeaacqWGJbWya0GaeyyeIuoaaSqaaiabdMgaPjabg2da9iabigdaXaqaaiabdkhaYbqdcqGHris5aOGaeiOla4caaa@4C49@

The relationship between *T *and *H*_2 _is described by a constant, the total number of interactions (*m*), as *T *= *m*·ln *m *- *m*·*H*_2_. Consequently, both methods yield exactly the same *p*-values.

### Relationship between *d*_*i *_and *H*_2_

In the following we derive the relationship between the individual levels of specialization (*d*_*i*_) and the community level (*H*_2_). The non-standardized Kullback-Leibler distance for row *i *can be rewritten as

di=∑j=1c(p′ij⋅ln⁡p′ijqj)=∑j=1c(pijqi⋅ln⁡pijqi⋅qj),
 MathType@MTEF@5@5@+=feaafiart1ev1aaatCvAUfKttLearuWrP9MDH5MBPbIqV92AaeXatLxBI9gBaebbnrfifHhDYfgasaacH8akY=wiFfYdH8Gipec8Eeeu0xXdbba9frFj0=OqFfea0dXdd9vqai=hGuQ8kuc9pgc9s8qqaq=dirpe0xb9q8qiLsFr0=vr0=vr0dc8meaabaqaciaacaGaaeqabaqabeGadaaakeaacqWGKbazdaWgaaWcbaGaemyAaKgabeaakiabg2da9maaqahabaWaaeWaceaacuWGWbaCgaqbamaaBaaaleaacqWGPbqAcqWGQbGAaeqaaOGaeyyXICTagiiBaWMaeiOBa42aaSaaaeaacuWGWbaCgaqbamaaBaaaleaacqWGPbqAcqWGQbGAaeqaaaGcbaGaemyCae3aaSbaaSqaaiabdQgaQbqabaaaaaGccaGLOaGaayzkaaaaleaacqWGQbGAcqGH9aqpcqaIXaqmaeaacqWGJbWya0GaeyyeIuoakiabg2da9maaqahabaWaaeWaceaadaWcaaqaaiabdchaWnaaBaaaleaacqWGPbqAcqWGQbGAaeqaaaGcbaGaemyCae3aaSbaaSqaaiabdMgaPbqabaaaaOGaeyyXICTagiiBaWMaeiOBa42aaSaaaeaacqWGWbaCdaWgaaWcbaGaemyAaKMaemOAaOgabeaaaOqaaiabdghaXnaaBaaaleaacqWGPbqAaeqaaOGaeyyXICTaemyCae3aaSbaaSqaaiabdQgaQbqabaaaaaGccaGLOaGaayzkaaaaleaacqWGQbGAcqGH9aqpcqaIXaqmaeaacqWGJbWya0GaeyyeIuoakiabcYcaSaaa@6D6E@

because pij=aijAi⋅Aim=p′ij.qi
 MathType@MTEF@5@5@+=feaafiart1ev1aaatCvAUfKttLearuWrP9MDH5MBPbIqV92AaeXatLxBI9gBaebbnrfifHhDYfgasaacH8akY=wiFfYdH8Gipec8Eeeu0xXdbba9frFj0=OqFfea0dXdd9vqai=hGuQ8kuc9pgc9s8qqaq=dirpe0xb9q8qiLsFr0=vr0=vr0dc8meaabaqaciaacaGaaeqabaqabeGadaaakeaacqWGWbaCdaWgaaWcbaGaemyAaKMaemOAaOgabeaakiabg2da9maalaaabaGaemyyae2aaSbaaSqaaiabdMgaPjabdQgaQbqabaaakeaacqWGbbqqdaWgaaWcbaGaemyAaKgabeaaaaGccqGHflY1daWcaaqaaiabdgeabnaaBaaaleaacqWGPbqAaeqaaaGcbaGaemyBa0gaaiabg2da9iqbdchaWzaafaWaaSbaaSqaaiabdMgaPjabdQgaQbqabaGccqGGUaGlcqWGXbqCdaWgaaWcbaGaemyAaKgabeaaaaa@4886@.

The weighted mean of *d*_*i *_for all *i *rows (each row weighted by *q*_*i*_) yields

〈di〉=∑i=1r(di⋅qi)=∑i=1r∑j=1c(pij⋅ln⁡pijqi⋅qj)=∑i=1r∑j=1c(pij⋅ln⁡pij)−∑i=1r∑j=1c(pij⋅ln⁡qi)−∑i=1r∑j=1c(pij⋅ln⁡qj)=∑i=1r∑j=1c(pij⋅ln⁡pij)−∑i=1r(qi⋅ln⁡qi)−∑j=1c(qj⋅ln⁡qj),
 MathType@MTEF@5@5@+=feaafiart1ev1aaatCvAUfKttLearuWrP9MDH5MBPbIqV92AaeXatLxBI9gBaebbnrfifHhDYfgasaacH8akY=wiFfYdH8Gipec8Eeeu0xXdbba9frFj0=OqFfea0dXdd9vqai=hGuQ8kuc9pgc9s8qqaq=dirpe0xb9q8qiLsFr0=vr0=vr0dc8meaabaqaciaacaGaaeqabaqabeGadaaakeaafaqadeWabaaabaGaeyykJeUaemizaq2aaSbaaSqaaiabdMgaPbqabaGccqGHQms8cqGH9aqpdaaeWbqaamaabmGabaGaemizaq2aaSbaaSqaaiabdMgaPbqabaGccqGHflY1cqWGXbqCdaWgaaWcbaGaemyAaKgabeaaaOGaayjkaiaawMcaaaWcbaGaemyAaKMaeyypa0JaeGymaedabaGaemOCaihaniabggHiLdGccqGH9aqpdaaeWbqaamaaqahabaWaaeWaceaacqWGWbaCdaWgaaWcbaGaemyAaKMaemOAaOgabeaakiabgwSixlGbcYgaSjabc6gaUnaalaaabaGaemiCaa3aaSbaaSqaaiabdMgaPjabdQgaQbqabaaakeaacqWGXbqCdaWgaaWcbaGaemyAaKgabeaakiabgwSixlabdghaXnaaBaaaleaacqWGQbGAaeqaaaaaaOGaayjkaiaawMcaaaWcbaGaemOAaOMaeyypa0JaeGymaedabaGaem4yamganiabggHiLdaaleaacqWGPbqAcqGH9aqpcqaIXaqmaeaacqWGYbGCa0GaeyyeIuoaaOqaaiabg2da9maaqahabaWaaabCaeaadaqadiqaaiabdchaWnaaBaaaleaacqWGPbqAcqWGQbGAaeqaaOGaeyyXICTagiiBaWMaeiOBa4MaemiCaa3aaSbaaSqaaiabdMgaPjabdQgaQbqabaaakiaawIcacaGLPaaaaSqaaiabdQgaQjabg2da9iabigdaXaqaaiabdogaJbqdcqGHris5aaWcbaGaemyAaKMaeyypa0JaeGymaedabaGaemOCaihaniabggHiLdGccqGHsisldaaeWbqaamaaqahabaWaaeWaceaacqWGWbaCdaWgaaWcbaGaemyAaKMaemOAaOgabeaakiabgwSixlGbcYgaSjabc6gaUjabdghaXnaaBaaaleaacqWGPbqAaeqaaaGccaGLOaGaayzkaaaaleaacqWGQbGAcqGH9aqpcqaIXaqmaeaacqWGJbWya0GaeyyeIuoaaSqaaiabdMgaPjabg2da9iabigdaXaqaaiabdkhaYbqdcqGHris5aOGaeyOeI0YaaabCaeaadaaeWbqaamaabmGabaGaemiCaa3aaSbaaSqaaiabdMgaPjabdQgaQbqabaGccqGHflY1cyGGSbaBcqGGUbGBcqWGXbqCdaWgaaWcbaGaemOAaOgabeaaaOGaayjkaiaawMcaaaWcbaGaemOAaOMaeyypa0JaeGymaedabaGaem4yamganiabggHiLdaaleaacqWGPbqAcqGH9aqpcqaIXaqmaeaacqWGYbGCa0GaeyyeIuoaaOqaaiabg2da9maaqahabaWaaabCaeaadaqadiqaaiabdchaWnaaBaaaleaacqWGPbqAcqWGQbGAaeqaaOGaeyyXICTagiiBaWMaeiOBa4MaemiCaa3aaSbaaSqaaiabdMgaPjabdQgaQbqabaaakiaawIcacaGLPaaaaSqaaiabdQgaQjabg2da9iabigdaXaqaaiabdogaJbqdcqGHris5aOGaeyOeI0YaaabCaeaadaqadiqaaiabdghaXnaaBaaaleaacqWGPbqAaeqaaOGaeyyXICTagiiBaWMaeiOBa4MaemyCae3aaSbaaSqaaiabdMgaPbqabaaakiaawIcacaGLPaaaaSqaaiabdMgaPjabg2da9iabigdaXaqaaiabdkhaYbqdcqGHris5aOGaeyOeI0YaaabCaeaadaqadiqaaiabdghaXnaaBaaaleaacqWGQbGAaeqaaOGaeyyXICTagiiBaWMaeiOBa4MaemyCae3aaSbaaSqaaiabdQgaQbqabaaakiaawIcacaGLPaaaaSqaaiabdQgaQjabg2da9iabigdaXaqaaiabdogaJbqdcqGHris5aOGaeiilaWcaleaacqWGPbqAcqGH9aqpcqaIXaqmaeaacqWGYbGCa0GaeyyeIuoaaaaaaa@0B60@

since qi=∑j=1cpij
 MathType@MTEF@5@5@+=feaafiart1ev1aaatCvAUfKttLearuWrP9MDH5MBPbIqV92AaeXatLxBI9gBaebbnrfifHhDYfgasaacH8akY=wiFfYdH8Gipec8Eeeu0xXdbba9frFj0=OqFfea0dXdd9vqai=hGuQ8kuc9pgc9s8qqaq=dirpe0xb9q8qiLsFr0=vr0=vr0dc8meaabaqaciaacaGaaeqabaqabeGadaaakeaacqWGXbqCdaWgaaWcbaGaemyAaKgabeaakiabg2da9maaqahabaGaemiCaa3aaSbaaSqaaiabdMgaPjabdQgaQbqabaaabaGaemOAaOMaeyypa0JaeGymaedabaGaem4yamganiabggHiLdaaaa@3BD4@ and qj=∑i=1rpij
 MathType@MTEF@5@5@+=feaafiart1ev1aaatCvAUfKttLearuWrP9MDH5MBPbIqV92AaeXatLxBI9gBaebbnrfifHhDYfgasaacH8akY=wiFfYdH8Gipec8Eeeu0xXdbba9frFj0=OqFfea0dXdd9vqai=hGuQ8kuc9pgc9s8qqaq=dirpe0xb9q8qiLsFr0=vr0=vr0dc8meaabaqaciaacaGaaeqabaqabeGadaaakeaacqWGXbqCdaWgaaWcbaGaemOAaOgabeaakiabg2da9maaqahabaGaemiCaa3aaSbaaSqaaiabdMgaPjabdQgaQbqabaaabaGaemyAaKMaeyypa0JaeGymaedabaGaemOCaihaniabggHiLdaaaa@3BF2@

While the first summand in the final equation for <*d*_*i*_> equals -*H*_2_, the remaining two summands correspond to the maximum entropy *H*_2max_, because

H2max⁡=−∑i=1r∑j=1c(qiqj⋅ln⁡qiqj)=−∑i=1r∑j=1c(qiqj⋅ln⁡qi)−∑i=1r∑j=1c(qiqj⋅ln⁡qj)=−∑i=1r(qi⋅ln⁡qi)−∑j=1c(qj⋅ln⁡qj).
 MathType@MTEF@5@5@+=feaafiart1ev1aaatCvAUfKttLearuWrP9MDH5MBPbIqV92AaeXatLxBI9gBaebbnrfifHhDYfgasaacH8akY=wiFfYdH8Gipec8Eeeu0xXdbba9frFj0=OqFfea0dXdd9vqai=hGuQ8kuc9pgc9s8qqaq=dirpe0xb9q8qiLsFr0=vr0=vr0dc8meaabaqaciaacaGaaeqabaqabeGadaaakeaafaqadeGabaaabaGaemisaG0aaSbaaSqaaiabikdaYiGbc2gaTjabcggaHjabcIha4bqabaGccqGH9aqpcqGHsisldaaeWbqaamaaqahabaWaaeWaceaacqWGXbqCdaWgaaWcbaGaemyAaKgabeaakiabdghaXnaaBaaaleaacqWGQbGAaeqaaOGaeyyXICTagiiBaWMaeiOBa4MaemyCae3aaSbaaSqaaiabdMgaPbqabaGccqWGXbqCdaWgaaWcbaGaemOAaOgabeaaaOGaayjkaiaawMcaaaWcbaGaemOAaOMaeyypa0JaeGymaedabaGaem4yamganiabggHiLdaaleaacqWGPbqAcqGH9aqpcqaIXaqmaeaacqWGYbGCa0GaeyyeIuoakiabg2da9iabgkHiTmaaqahabaWaaabCaeaadaqadiqaaiabdghaXnaaBaaaleaacqWGPbqAaeqaaOGaemyCae3aaSbaaSqaaiabdQgaQbqabaGccqGHflY1cyGGSbaBcqGGUbGBcqWGXbqCdaWgaaWcbaGaemyAaKgabeaaaOGaayjkaiaawMcaaaWcbaGaemOAaOMaeyypa0JaeGymaedabaGaem4yamganiabggHiLdaaleaacqWGPbqAcqGH9aqpcqaIXaqmaeaacqWGYbGCa0GaeyyeIuoakiabgkHiTmaaqahabaWaaabCaeaadaqadiqaaiabdghaXnaaBaaaleaacqWGPbqAaeqaaOGaemyCae3aaSbaaSqaaiabdQgaQbqabaGccqGHflY1cyGGSbaBcqGGUbGBcqWGXbqCdaWgaaWcbaGaemOAaOgabeaaaOGaayjkaiaawMcaaaWcbaGaemOAaOMaeyypa0JaeGymaedabaGaem4yamganiabggHiLdaaleaacqWGPbqAcqGH9aqpcqaIXaqmaeaacqWGYbGCa0GaeyyeIuoaaOqaaiabg2da9iabgkHiTmaaqahabaWaaeWaceaacqWGXbqCdaWgaaWcbaGaemyAaKgabeaakiabgwSixlGbcYgaSjabc6gaUjabdghaXnaaBaaaleaacqWGPbqAaeqaaaGccaGLOaGaayzkaaaaleaacqWGPbqAcqGH9aqpcqaIXaqmaeaacqWGYbGCa0GaeyyeIuoakiabgkHiTmaaqahabaWaaeWaceaacqWGXbqCdaWgaaWcbaGaemOAaOgabeaakiabgwSixlGbcYgaSjabc6gaUjabdghaXnaaBaaaleaacqWGQbGAaeqaaaGccaGLOaGaayzkaaaaleaacqWGQbGAcqGH9aqpcqaIXaqmaeaacqWGJbWya0GaeyyeIuoakiabc6caUaaaaaa@BE58@

Therefore,

<*d*_*i*_> = *H*_2max _-*H*_2_.

The same calculation applies for <*d*_*j*_>, thus <*d*_*i*_> = <*d*_*j*_>. Consequently, the degree of specialization of the entire network (corresponding to the deviation of the network-wide entropy from its maximum value) equals the weighted sum of the specialization of its elements (species).

### Properties of alternative niche breadth measures

The standardized Hurlbert's (*B*') and Smith's (*FT*) measure can be applied widely for niche breadth analysis [21,22,24]. In this context, the Kullback-Leibler distance (*d*) can be viewed as a modified Shannon-Wiener measure of niche breadth that accounts for niche availabilities. Like the Kullback-Leibler distance, both *B*' and *FT *compare the proportional distribution of individuals (*p*) to the proportional resource availability (*q*) (here: partner availability). For a certain species *i*, the two measures are in our notation:

B′i=Bi−qmin⁡1−qmin⁡, with Bi=1∑j=1c(pij2/qj), and FTi=∑j=1c(pij⋅qj).
 MathType@MTEF@5@5@+=feaafiart1ev1aaatCvAUfKttLearuWrP9MDH5MBPbIqV92AaeXatLxBI9gBaebbnrfifHhDYfgasaacH8akY=wiFfYdH8Gipec8Eeeu0xXdbba9frFj0=OqFfea0dXdd9vqai=hGuQ8kuc9pgc9s8qqaq=dirpe0xb9q8qiLsFr0=vr0=vr0dc8meaabaqaciaacaGaaeqabaqabeGadaaakeaacuWGcbGqgaqbamaaBaaaleaacqWGPbqAaeqaaOGaeyypa0ZaaSaaaeaacqWGcbGqdaWgaaWcbaGaemyAaKgabeaakiabgkHiTiabdghaXnaaBaaaleaacyGGTbqBcqGGPbqAcqGGUbGBaeqaaaGcbaGaeGymaeJaeyOeI0IaemyCae3aaSbaaSqaaiGbc2gaTjabcMgaPjabc6gaUbqabaaaaOGaeiilaWIaeeiiaaIaee4DaCNaeeyAaKMaeeiDaqNaeeiAaGMaeeiiaaIaemOqai0aaSbaaSqaaiabdMgaPbqabaGccqGH9aqpdaWcaaqaaiabigdaXaqaamaaqahabaWaaeWaceaadaWcgaqaaiabdchaWnaaDaaaleaacqWGPbqAcqWGQbGAaeaacqaIYaGmaaaakeaacqWGXbqCdaWgaaWcbaGaemOAaOgabeaaaaaakiaawIcacaGLPaaaaSqaaiabdQgaQjabg2da9iabigdaXaqaaiabdogaJbqdcqGHris5aaaakiabcYcaSiabbccaGiabbggaHjabb6gaUjabbsgaKjabbccaGiabdAeagjabdsfaunaaBaaaleaacqWGPbqAaeqaaOGaeyypa0ZaaabCaeaadaqadiqaamaakaaabaGaemiCaa3aaSbaaSqaaiabdMgaPjabdQgaQbqabaGccqGHflY1cqWGXbqCdaWgaaWcbaGaemOAaOgabeaaaeqaaaGccaGLOaGaayzkaaaaleaacqWGQbGAcqGH9aqpcqaIXaqmaeaacqWGJbWya0GaeyyeIuoakiabc6caUaaa@7D29@

Each *p*'_*ij *_is the proportion of the number of interactions in relation to the respective row total, and *q*_*j *_is the proportion of all interactions by partner *j *in relation to the total number of interactions. Thus,

∑j=1cp′ij=1,∑j=1cqj=1, and qmin⁡=min⁡(qj).
 MathType@MTEF@5@5@+=feaafiart1ev1aaatCvAUfKttLearuWrP9MDH5MBPbIqV92AaeXatLxBI9gBaebbnrfifHhDYfgasaacH8akY=wiFfYdH8Gipec8Eeeu0xXdbba9frFj0=OqFfea0dXdd9vqai=hGuQ8kuc9pgc9s8qqaq=dirpe0xb9q8qiLsFr0=vr0=vr0dc8meaabaqaciaacaGaaeqabaqabeGadaaakeaadaaeWbqaaiqbdchaWzaafaWaaSbaaSqaaiabdMgaPjabdQgaQbqabaaabaGaemOAaOMaeyypa0JaeGymaedabaGaem4yamganiabggHiLdGccqGH9aqpcqaIXaqmcqGGSaaldaaeWbqaaiabdghaXnaaBaaaleaacqWGQbGAaeqaaaqaaiabdQgaQjabg2da9iabigdaXaqaaiabdogaJbqdcqGHris5aOGaeyypa0JaeGymaeJaeiilaWIaeeiiaaIaeeyyaeMaeeOBa4MaeeizaqMaeeiiaaIaemyCae3aaSbaaSqaaiGbc2gaTjabcMgaPjabc6gaUbqabaGccqGH9aqpcyGGTbqBcqGGPbqAcqGGUbGBdaqadiqaaiabdghaXnaaBaaaleaacqWGQbGAaeqaaaGccaGLOaGaayzkaaGaeiOla4caaa@5D4C@

Both the standardized Hurlbert's (*B*') and Smith's (*FT*) measure range between 0 for the most specialized case to 1.0 for extreme generalization (broadest niche). In the context of niche breadth, it has been shown that the Shannon-Wiener measure is most sensitive, while Hurlbert's and particularly Smith's measure are less sensitive for the selection of rare resources [21] (see also [20]).

For the application in network analyses, however, both *B*' and *FT *may show some undesired properties. Generally, *B*', *FT *and *d*' are reasonably well correlated with each other across the species within a network (e.g., *r*_*s *_= -0.49 between *d*' and *B*', and *r*_*s *_= -0.36 between *d*' and *FT *for the 90 pollinators in the network of Vázquez and Simberloff [33], both *p *< 0.001). However, differences with *d*' are substantial when a highly specialized species interacts largely exclusively with a specialized partner, e.g. a specialized pollinator with a plant that is almost exclusively pollinated by this one. Imagine a scenario where one exclusive interaction occurs between a plant species and a pollinator species in a 3 × 3 matrix (Table 2). If the interaction between pollinator sp. 3 and plant sp. 3 is only infrequent (e.g. *a*_*33 *_= 1), all indices show a high degree of specialization (*d*' = 1.0, *B*' = 0, *FT *= 0.14) for both partners. However, as the number of exclusive interactions (*a*_*33*_) increases, the values for both *B*' and *FT *of pollinator sp. 3 and plant sp. 3 show a highly undesired change towards generalization, although a higher *a*_*33 *_is intuitively considered as extreme specialization (e.g., for *a*_*33 *_= 50 the values for pollinator sp. 3 are *B*' = 0.31 and *FT *= 0.70), while only *d*' remains unaffected (*d*' = 1.0). *FT *is always larger than zero, and *B*' becomes larger than zero when the specialists interact more frequently than one of the other partners, thus when *q*_*j *_> min(*q*_1_, *q*_2_, ... *q*_*c*_). Both *FT *and *B*' approach a value of 1.0 (maximum generalization) for very large *a*_*33*_. This undesired effect of *FT *and *B*' is not restricted to completely exclusive interactions between two partners.

### Simulation of sampling effort and matrix architecture

Two published plant-pollinator networks were selected to investigate the behavior of different specialization measures [32,33]. Both articles use their observed interaction matrices as a model to discuss network properties based on the number of links per pollinator or plant species, allowing a comparison of conclusions drawn. Both networks may be compared as they comprise relatively large datasets from temperate ecosystems, reporting interaction frequencies between plants and their floral visitors: the British meadow community studied by Memmott [32] involved 79 pollinator and 25 plant species (2183 pollinator visits observed), the forests in Argentina studied by Vázquez and Simberloff [33] involved 90 pollinator and 14 plant species (5285 visits). The datasets can be obtained from the Interaction Web Database [53]. We simulated a decreased sampling intensity in both networks using a rarefaction method in order to investigate how sampling effort affects the estimation of specialization indices. Real association matrices were reduced by randomly extracting interactions, e.g. from the total of *m *= 2183 visits in Memmott's web down to *m *= 5 visits (in steps of five, repeated ten times for each *m*).

In order to compare the null model characteristics of the specialization measures, we simulated artificial matrices with randomly associated partners and plotted the indices against an increasing number of partners and/or total number of interactions. We assumed that the total frequency of participating species approximates a lognormal distribution, which is typical for biological communities [21,22,24]. All row and column totals were randomly generated from a lognormal distribution (*μ *= 50, *∑*= 1) that was scaled to the desired total number of interactions. Ten different combinations of row and column totals were obtained for each matrix size and taken as template to randomly associate the partners five times, thus each matrix size was represented by 50 random associations.

## Authors' contributions

NB1 conceived of the study and all authors (NB1, FM, NB2) were involved in designing the methods, analyses, interpretation and drafting the manuscript.

## Figures and Tables

**Table 1:** Elements in a species association matrix. Interaction frequencies (*a*_ij_) between *c *animal and *r *plant species and their respective totals (rows:*A*_i_, columns: *A*_j_, total elements: *m*).

	Animal sp.1	sp. 2	...	sp. *c*	*Total*
Plant sp. 1	*a*_11_	*a*_12_	...	a_1*c*_	Ai=1=∑j=1ca1j MathType@MTEF@5@5@+=feaafiart1ev1aaatCvAUfKttLearuWrP9MDH5MBPbIqV92AaeXatLxBI9gBaebbnrfifHhDYfgasaacH8akY=wiFfYdH8Gipec8Eeeu0xXdbba9frFj0=OqFfea0dXdd9vqai=hGuQ8kuc9pgc9s8qqaq=dirpe0xb9q8qiLsFr0=vr0=vr0dc8meaabaqaciaacaGaaeqabaqabeGadaaakeaacqWGbbqqdaWgaaWcbaGaemyAaKMaeyypa0JaeGymaedabeaakiabg2da9maaqahabaGaemyyae2aaSbaaSqaaiabigdaXiabdQgaQbqabaaabaGaemOAaOMaeyypa0JaeGymaedabaGaem4yamganiabggHiLdaaaa@3CE1@
sp. 2	a_21_	*a*_22_	...	a_2*c*_	Ai=2=∑j=1ca2j MathType@MTEF@5@5@+=feaafiart1ev1aaatCvAUfKttLearuWrP9MDH5MBPbIqV92AaeXatLxBI9gBaebbnrfifHhDYfgasaacH8akY=wiFfYdH8Gipec8Eeeu0xXdbba9frFj0=OqFfea0dXdd9vqai=hGuQ8kuc9pgc9s8qqaq=dirpe0xb9q8qiLsFr0=vr0=vr0dc8meaabaqaciaacaGaaeqabaqabeGadaaakeaacqWGbbqqdaWgaaWcbaGaemyAaKMaeyypa0JaeGOmaidabeaakiabg2da9maaqahabaGaemyyae2aaSbaaSqaaiabikdaYiabdQgaQbqabaaabaGaemOAaOMaeyypa0JaeGymaedabaGaem4yamganiabggHiLdaaaa@3CE5@
...	...	...	...	...	...
sp. *r*	*a*_*r*1_	*a*_*r*2_	...	a_*rc*_	Ai=r=∑j=1carj MathType@MTEF@5@5@+=feaafiart1ev1aaatCvAUfKttLearuWrP9MDH5MBPbIqV92AaeXatLxBI9gBaebbnrfifHhDYfgasaacH8akY=wiFfYdH8Gipec8Eeeu0xXdbba9frFj0=OqFfea0dXdd9vqai=hGuQ8kuc9pgc9s8qqaq=dirpe0xb9q8qiLsFr0=vr0=vr0dc8meaabaqaciaacaGaaeqabaqabeGadaaakeaacqWGbbqqdaWgaaWcbaGaemyAaKMaeyypa0JaemOCaihabeaakiabg2da9maaqahabaGaemyyae2aaSbaaSqaaiabdkhaYjabdQgaQbqabaaabaGaemOAaOMaeyypa0JaeGymaedabaGaem4yamganiabggHiLdaaaa@3DDB@

*Total*	Aj=1=∑i=1rai1 MathType@MTEF@5@5@+=feaafiart1ev1aaatCvAUfKttLearuWrP9MDH5MBPbIqV92AaeXatLxBI9gBaebbnrfifHhDYfgasaacH8akY=wiFfYdH8Gipec8Eeeu0xXdbba9frFj0=OqFfea0dXdd9vqai=hGuQ8kuc9pgc9s8qqaq=dirpe0xb9q8qiLsFr0=vr0=vr0dc8meaabaqaciaacaGaaeqabaqabeGadaaakeaacqWGbbqqdaWgaaWcbaGaemOAaOMaeyypa0JaeGymaedabeaakiabg2da9maaqahabaGaemyyae2aaSbaaSqaaiabdMgaPjabigdaXaqabaaabaGaemyAaKMaeyypa0JaeGymaedabaGaemOCaihaniabggHiLdaaaa@3CFD@	Aj=2=∑i=1rai2 MathType@MTEF@5@5@+=feaafiart1ev1aaatCvAUfKttLearuWrP9MDH5MBPbIqV92AaeXatLxBI9gBaebbnrfifHhDYfgasaacH8akY=wiFfYdH8Gipec8Eeeu0xXdbba9frFj0=OqFfea0dXdd9vqai=hGuQ8kuc9pgc9s8qqaq=dirpe0xb9q8qiLsFr0=vr0=vr0dc8meaabaqaciaacaGaaeqabaqabeGadaaakeaacqWGbbqqdaWgaaWcbaGaemOAaOMaeyypa0JaeGOmaidabeaakiabg2da9maaqahabaGaemyyae2aaSbaaSqaaiabdMgaPjabikdaYaqabaaabaGaemyAaKMaeyypa0JaeGymaedabaGaemOCaihaniabggHiLdaaaa@3D01@	...	Aj=c=∑i=1raic MathType@MTEF@5@5@+=feaafiart1ev1aaatCvAUfKttLearuWrP9MDH5MBPbIqV92AaeXatLxBI9gBaebbnrfifHhDYfgasaacH8akY=wiFfYdH8Gipec8Eeeu0xXdbba9frFj0=OqFfea0dXdd9vqai=hGuQ8kuc9pgc9s8qqaq=dirpe0xb9q8qiLsFr0=vr0=vr0dc8meaabaqaciaacaGaaeqabaqabeGadaaakeaacqWGbbqqdaWgaaWcbaGaemOAaOMaeyypa0Jaem4yamgabeaakiabg2da9maaqahabaGaemyyae2aaSbaaSqaaiabdMgaPjabdogaJbqabaaabaGaemyAaKMaeyypa0JaeGymaedabaGaemOCaihaniabggHiLdaaaa@3DBB@	m=∑i=1r∑j=1caij MathType@MTEF@5@5@+=feaafiart1ev1aaatCvAUfKttLearuWrP9MDH5MBPbIqV92AaeXatLxBI9gBaebbnrfifHhDYfgasaacH8akY=wiFfYdH8Gipec8Eeeu0xXdbba9frFj0=OqFfea0dXdd9vqai=hGuQ8kuc9pgc9s8qqaq=dirpe0xb9q8qiLsFr0=vr0=vr0dc8meaabaqaciaacaGaaeqabaqabeGadaaakeaacqWGTbqBcqGH9aqpdaaeWbqaamaaqahabaGaemyyae2aaSbaaSqaaiabdMgaPjabdQgaQbqabaaabaGaemOAaOMaeyypa0JaeGymaedabaGaem4yamganiabggHiLdaaleaacqWGPbqAcqGH9aqpcqaIXaqmaeaacqWGYbGCa0GaeyyeIuoaaaa@411D@

**Table 2:** Association matrix example. Fictive association matrix between three pollinator species and three plant species. Numbers in each cell are counts of interaction frequencies.

	Pollinator sp. 1	Pollinator sp. 2	**Pollinator sp. 3**
Plant sp. 1	21	5	0
Plant sp. 2	23	4	0
**Plant sp. 3**	0	0	***a***_***33***_
